# High-Speed FBG Interrogation for Multi-Point Lithium-Ion Battery Temperature Monitoring Using a REC-DFB Laser Array

**DOI:** 10.3390/s26196215

**Published:** 2026-09-30

**Authors:** Yi Yang, Jianyu Yu, Qi Zhou, Kaichuan Xu, Pan Dai, Zhenzhen Xu, Xiangfei Chen

**Affiliations:** 1 State Grid Jiangsu Electric Power Co., Ltd., Nanjing 210093, China; yangydky@js.sgcc.com.cn (Y.Y.); yujy@js.sgcc.com.cn (J.Y.); zhouq19@js.sgcc.com.cn (Q.Z.); 2 Key Laboratory of Intelligent Optical Sensing and Manipulation of the Ministry of Education, Engineering Research Center of Precision Photonics Integration and System Application of the Ministry of Education, National Laboratory of Solid State Microstructures, College of Engineering and Applied Sciences, Institute of Optical Communication Engineering, Nanjing University-Tongding Joint Lab for Large-Scale Photonic Integrated Circuits, Nanjing University, Nanjing 210093, China; 602023340022@smail.nju.edu.cn (K.X.); pdai@nju.edu.cn (P.D.); 2025101570@niit.edu.cn (Z.X.)

**Keywords:** lithium-ion battery, fiber Bragg grating (FBG), temperature monitoring, REC-DFB laser array, high-speed interrogation, multi-point sensing, strain isolation

## Abstract

Lithium-ion batteries can undergo rapid and spatially nonuniform temperature variations during high-rate operation, requiring multi-point temperature monitoring with high temporal resolution for effective thermal management. This work presents a high-speed fiber Bragg grating (FBG) temperature monitoring system based on a reconstruction-equivalent-chirp distributed feedback (REC-DFB) laser array. The developed 24-channel REC-DFB laser array, integrated with an on-chip optical combiner and semiconductor optical amplifier, achieves a continuous wavelength-sweeping range of approximately 50 nm, with a tuning range of approximately 2.4 nm per channel and channel power equalization. To suppress temperature–strain cross-sensitivity caused by battery deformation, strain-isolated FBG temperature sensors with polyimide packaging are developed, exhibiting sensitivities of 9.7–10.4 pm/°C over 20–80 °C with coefficients of determination (R^2^) above 0.998. Battery experiments are performed at discharge rates up to 4 C, with simultaneous FBG and thermocouple measurements at the top, middle, and bottom positions. The proposed system achieves an interrogation rate of 5 kHz and shows good agreement with thermocouples under slowly varying thermal conditions. During rapid thermal transients at 4 C, the peak temperature rises measured by the FBGs are 2.1–2.6 °C higher than those measured by the thermocouples, while a maximum temperature-rise difference of 5.98 °C is observed across the battery surface. These results demonstrate the capability of the proposed all-solid-state, mechanically scan-free system for high-speed, multi-point monitoring of dynamic and spatially nonuniform thermal behavior in lithium-ion batteries.

## 1. Introduction

Lithium-ion batteries (LIBs) are highly sensitive to operating temperature, which directly affects their safety, performance, and cycle life [1,2,3,4]. During high-rate charging and discharging, intensive electrochemical reactions and Joule heating can induce rapid and spatially nonuniform temperature rises across the battery surface. Accurate monitoring of these thermal variations is therefore essential for evaluating battery thermal states, optimizing thermal management, and providing early warning of abnormal heating. However, conventional battery management systems typically rely on a limited number of temperature sensors, making it difficult to simultaneously capture spatial temperature gradients and rapid thermal transients. High-speed, multi-point surface temperature monitoring is thus highly desirable for reliable LIB operation.

Thermocouples are widely used for battery temperature measurement because of their simplicity and low cost [5,6,7]. However, as electrical sensors, they are inherently conductive, susceptible to electromagnetic interference, and limited by thermal inertia. Fiber Bragg grating (FBG) sensors provide an attractive alternative owing to their electrical insulation, immunity to electromagnetic interference, compact size, low thermal mass, and wavelength-division multiplexing capability [8,9,10,11]. Multiple FBGs can be distributed over the battery surface to simultaneously monitor temperature evolution at different locations, offering significant advantages for characterizing spatial and dynamic thermal behavior.

Despite these advantages, the performance of FBG-based battery temperature monitoring systems is strongly dependent on the interrogation technique. Conventional FBG interrogators typically employ broadband light sources combined with optical spectrum analyzers [12,13] or tunable Fabry–Perot (F-P) filters [14,15,16]. Although these approaches provide effective wavelength interrogation, their scanning speed and, in mechanically tuned configurations, long-term stability can limit the temporal resolution of dynamic temperature measurements. Recent studies have explored tunable and interferometric structures for optical filtering and wavelength selection. Ramos-Gonzalez et al. developed a tunable F-P filter based on a silicon wafer for infrared gas sensing [17], while Bueno-Gasca et al. demonstrated a Michelson–F-P comb filter for a switchable linear fiber laser [18]. These studies highlight the potential of such structures for spectral selection. For multi-point FBG temperature monitoring, however, broad wavelength coverage and rapid, repeatable scanning are also required. These requirements motivate the development of a high-speed, mechanically scan-free interrogation source for dynamic temperature monitoring.

To address this limitation, a reconstruction-equivalent-chirp distributed feedback (REC-DFB) laser array is employed in this work as a high-speed swept-wavelength source for FBG interrogation. By sequentially tuning multiple DFB lasers through current modulation, broadband wavelength scanning can be achieved without mechanical tuning components. The all-solid-state architecture combines high scanning speed with stable operation, providing an effective platform for high-speed, multi-point FBG interrogation.

Another critical challenge in surface-mounted FBG temperature sensing is temperature–strain cross-sensitivity. During battery cycling, electrode expansion and contraction induce deformation of the battery casing, which can be transferred to a surface-mounted FBG through the adhesive layer. Consequently, the measured Bragg wavelength shift contains contributions from both temperature and mechanical strain, resulting in temperature measurement errors. To suppress this effect, a strain-isolated FBG temperature sensor with polyimide (PI) packaging is developed. The grating region is mechanically decoupled from external deformation, allowing the Bragg wavelength shift to predominantly reflect temperature variations without requiring additional reference gratings or complex temperature–strain decoupling algorithms.

This work combines a 24-channel REC-DFB laser array [19,20] with strain-isolated FBG sensors for multi-point battery surface-temperature monitoring. On-chip optical combining, SOA-based power equalization, and electronic wavelength scanning provide approximately 50 nm of wavelength coverage and a 5 kHz interrogation rate, while PI packaging reduces deformation-induced temperature–strain cross-sensitivity. Experiments with three FBGs and adjacent thermocouples characterize spatial and temporal temperature variations under different discharge rates. The main contribution is the integration of broadband, high-speed interrogation with strain-isolated sensing for battery temperature monitoring. The wavelength coverage offers potential for additional wavelength-multiplexed sensors, although larger sensor arrays and battery-module applications require further validation.

## 2. FBG Sensing Principle

**Figure 1:**
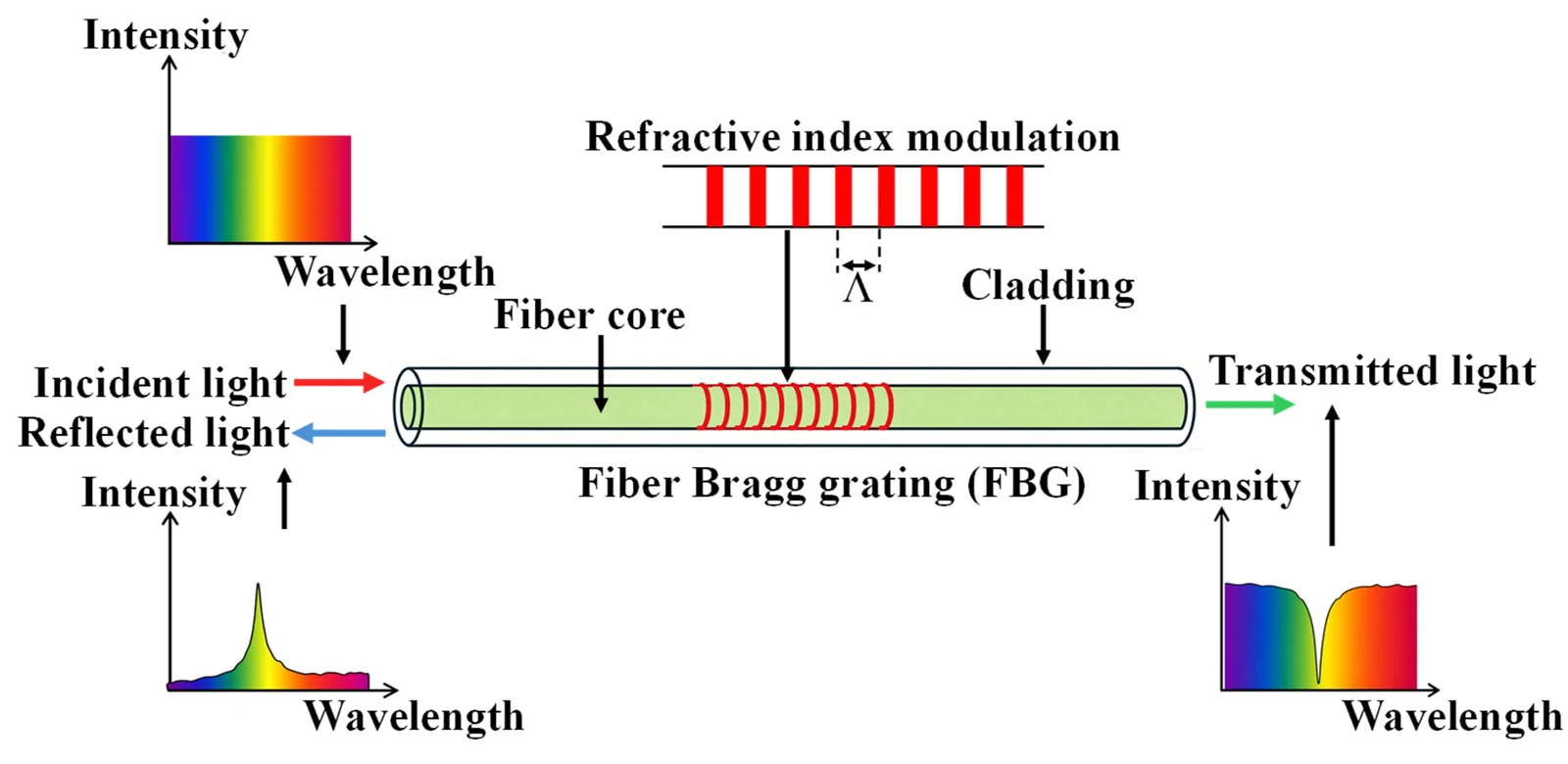
Schematic of the FBG sensing principle.

The sensing principle of an FBG is illustrated in Figure 1. Its central reflection wavelength, known as the Bragg wavelength, is expressed as [21,22]:

λ*_B_* = 2*n_eff_*Λ, (1)

where *n*_eff_ is the effective refractive index of the fundamental core mode and *Λ* is the grating period. Variations in temperature or strain alter *n*_eff_ and *Λ*, resulting in a shift in the Bragg wavelength. Therefore, external physical quantities can be measured by accurately interrogating the Bragg wavelength shift. When an FBG is subjected to both temperature changes and axial strain, the Bragg wavelength shift can be expressed as:

(2) $$\frac{\Delta \lambda_{B}}{\lambda_{B}}=(\alpha +\xi )\Delta T+(1-\rho_{e})\varepsilon ,$$

where *α* and *ξ* are the thermal expansion and thermo-optic coefficients of the fiber, respectively, *p*_e_ is the effective photoelastic coefficient, and *ΔT* and *ε* denote the temperature variation and axial strain, respectively. This also can be expressed as:

∆*λ_B_* = *K_T_*∆*T* + *K_ε_*∆*ε*, (3)

*K*_ε_ represents the strain sensitivity and *K*_T_ represents the temperature sensitivity. represents the strain sensitivity and K_T_ represents the temperature sensitivity. This relationship indicates that both temperature and strain contribute to the Bragg wavelength shift. Therefore, for surface temperature monitoring of lithium-ion batteries, the influence of mechanical strain on temperature measurements should be effectively suppressed.

In addition, simultaneous interrogation of multiple FBG sensors requires the wavelength coverage of the light source to encompass the reflection bands of all FBGs over the entire measurement range. Broadband wavelength scanning not only enables complete tracking of Bragg wavelength shifts but also facilitates multi-point sensing of FBGs with different center wavelengths through wavelength-division multiplexing. Therefore, a high-speed swept-wavelength source with broad spectral coverage is essential for multi-point dynamic temperature monitoring of lithium-ion batteries.

## 3. Design and Performance Characterization of the REC-DFB Laser Array

### 3.1. Design of the REC-DFB Laser Array

To address the limited scanning speed and long-term stability of conventional broadband sources, a self-developed REC-DFB laser array is employed as a broadband high-speed swept-wavelength source. The REC technique enables complex grating structures through micrometer-scale sampling modulation. Specifically, a uniform seed grating is fabricated by a single holographic exposure, followed by conventional photolithography to define the sampling pattern, thereby transferring the stringent control of the nanoscale grating period to the micrometer-scale sampling period. In the REC structure, the period of the +1st-order equivalent sub-grating, Λ_+1_, is given by [23]:

(4) $$\frac{1}{\Lambda_{+1}}=\frac{1}{\Lambda_{0}}+\frac{1}{P},$$

where Λ_0_ is the seed grating period and *P* is the sampling period. Therefore, the lasing wavelength can be precisely controlled by adjusting the micrometer-scale sampling period *P*, while maintaining an identical seed grating across the entire laser array. The relationship between the equivalent grating-period error and the sampling-period error is expressed as:

(5) $$\Delta \Lambda_{+1}=\frac{1}{{\left(\frac{P}{\Lambda_{0}}+1\right)}^{2}}\Delta P.$$

Since *P* ≫ Λ_0_, the REC technique transfers wavelength control from nanoscale grating fabrication to micrometer-scale photolithography, substantially reducing fabrication errors and improving wavelength uniformity and device yield, which facilitates the reliable fabrication of large-scale DFB laser arrays.

**Figure 2:**
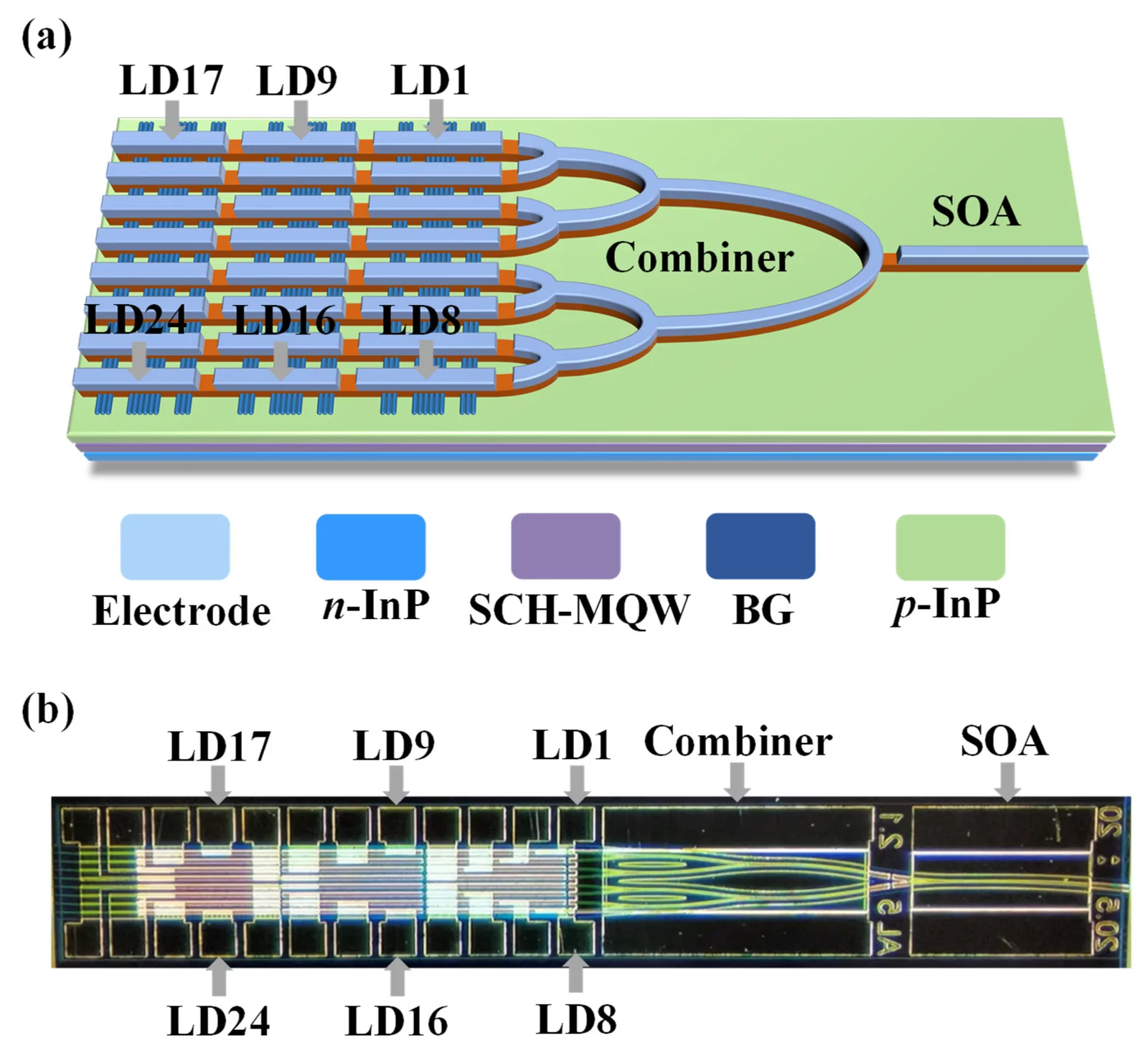
24-channel REC-DFB laser array chip: (**a**) schematic structure; (**b**) photograph of the fabricated chip.

Based on this principle, a 24-channel DFB laser array chip was designed, as illustrated in Figure 2a, with the fabricated device shown in Figure 2b. The InP-based chip monolithically integrates the DFB laser array, optical combining waveguides, and a semiconductor optical amplifier (SOA). The 24 DFB lasers are arranged in a staggered 8 × 3 configuration, with a designed channel spacing of 2.0 nm and an overall wavelength coverage of 1518–1568 nm. The staggered layout increases the physical separation between adjacent wavelength channels, thereby suppressing thermal crosstalk and ensuring stable single-longitudinal-mode operation. The outputs of the DFB lasers are combined into a single optical path through a three-stage on-chip Y-branch network and subsequently injected into the integrated SOA. The SOA provides both optical amplification and dynamic power equalization, compensating for combining losses, intrinsic channel-to-channel power variations, and wavelength-dependent gain through current control. This improves the spectral flatness of the broadband swept output and the signal-to-noise ratio of FBG interrogation. The fully solid-state architecture eliminates mechanical tuning components, providing a compact and stable platform for high-speed broadband wavelength scanning.

### 3.2. Test Results of the REC-DFB Laser Array

**Figure 3:**
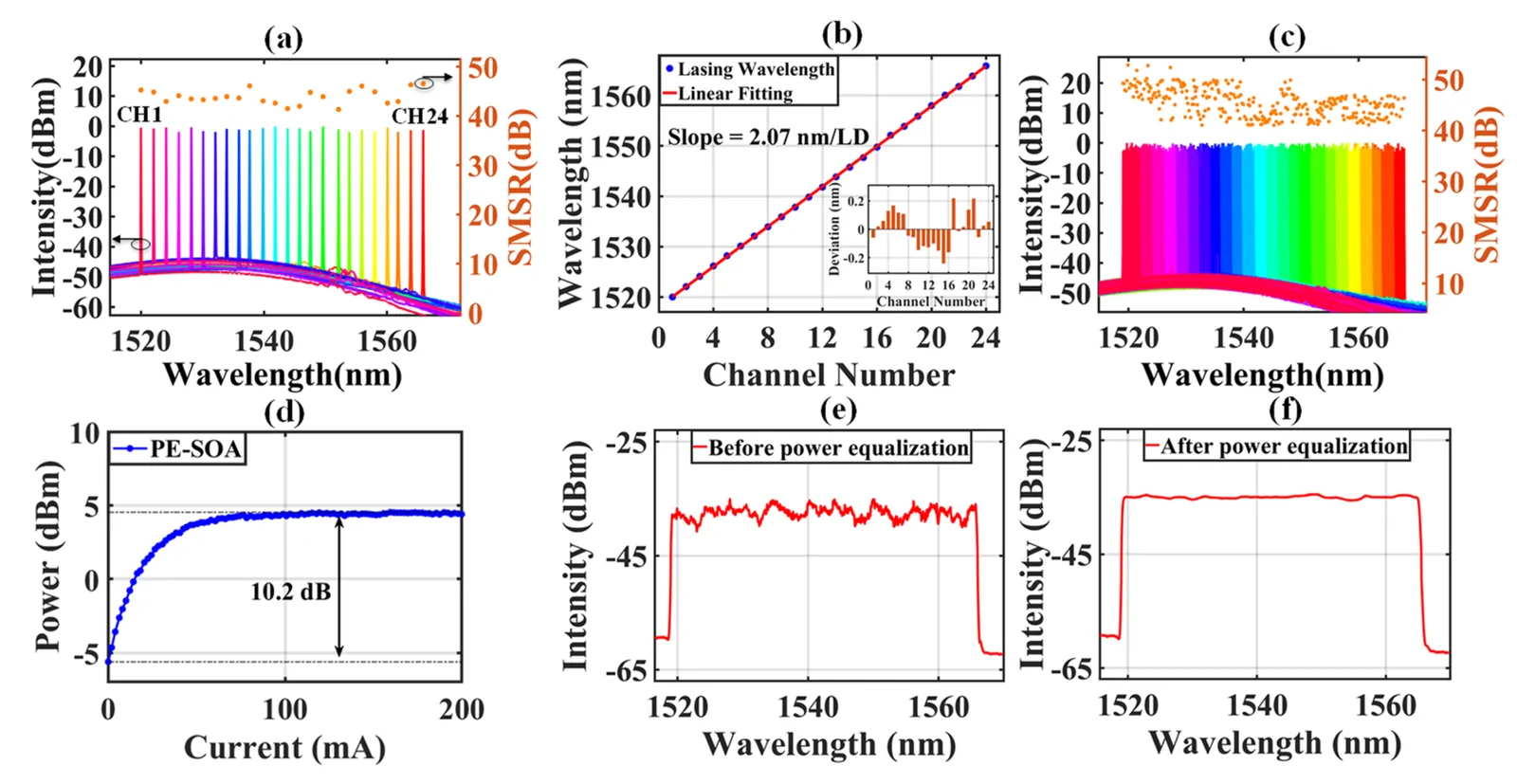
Spectral, wavelength-tuning, and power-equalization characteristics of the 24-channel REC-DFB laser array. (**a**) Measured output spectra of the 24 DFB channels. (**b**) Measured center wavelengths and linear fitting results. (**c**) Wavelength-tuning characteristics of the 24 DFB channels under different injection currents. (**d**) SOA output power as a function of drive current. (**e**) Broadband swept output with a fixed SOA current. (**f**) Broadband swept output after dynamic power equalization.

The output characteristics of the developed REC-DFB laser array were systematically characterized in terms of spectral performance, wavelength tunability, and SOA-based power regulation and equalization. For the static spectral measurements shown in Figure 3a, the drive currents of the DFB lasers, optical combining section, and SOA were set to 80,100, and 80 mA, respectively. All channels exhibit stable single-longitudinal-mode operation with side-mode suppression ratios (SMSRs) exceeding 38 dB. Figure 3b shows the measured center wavelengths and their linear fit. The fitted slope is 2.07 nm, corresponding to an average channel spacing of approximately 2.07 nm, in good agreement with the designed value of 2.0 nm. As shown in the inset, the maximum deviation of the measured center wavelengths from the linear fit is below 0.3 nm, demonstrating the precise wavelength control enabled by the REC technique.

The wavelength-tuning characteristics of the 24 DFB channels were further characterized by varying the injection current from 50 to 180 mA. As shown in Figure 3c, the center wavelength of each channel shifts continuously toward longer wavelengths as the injection current increases, yielding a tuning range of approximately 2.4 nm per channel. The tuning ranges of all 24 channels are summarized in Table 1. Adjacent channels exhibit wavelength overlaps of approximately 0.3–0.4 nm, preventing spectral gaps during channel switching and enabling continuous broadband wavelength scanning through spectral stitching.

The power-regulation capability of the integrated SOA was subsequently evaluated by varying its drive current from 0 to 200 mA in steps of 2 mA while maintaining constant laser input conditions. As shown in Figure 3d, the output power increases with the SOA current and gradually approaches saturation above approximately 120 mA, providing an overall output-power tuning range of approximately 10.2 dB. This result confirms that the integrated SOA provides sufficient gain tunability to compensate for on-chip combining losses and intrinsic power variations among the DFB channels.

During the broadband wavelength scanning process, the dynamic power-equalization capability of the SOA was further studied. The measurement results shown in Figure 3e were obtained before the balance was implemented. At this time, LD1–LD24 were successively tuned within the injection current range of 50–180 mA, while the driving currents of the optical combining section and the SOA remained at 100 mA and 80 mA, respectively, achieving continuous wavelength coverage on 24 channels. However, due to the inherent DFB output power, the on-chip combining loss in the chip, and the wavelength-dependent SOA gain differences, there were still significant power fluctuations. In the dynamic power equalization experiment shown in Figure 3f, to suppress these fluctuations, a feedback-based dynamic power equalization scheme was implemented. That is, based on the deviation between the measured output power and the target output power, the SOA driving currents under each wavelength were iteratively adjusted.

After dynamic equalization, the broadband swept output exhibits substantially improved power uniformity, as shown in Figure 3f. Compared with operation at a fixed SOA current, the equalized spectrum shows more uniform output power across the wavelength range, demonstrating effective compensation for channel-to-channel power variations and broadband gain fluctuations. These results confirm that the developed 24-channel REC-DFB laser array provides precise wavelength control, continuous broadband tunability, and effective dynamic power equalization, making it well suited as a high-speed and stable swept-wavelength source for FBG interrogation.

### 3.3. Wavelength Calibration and Correction of the REC-DFB Laser Array

Although the 24-channel REC-DFB laser array enables continuous broadband wavelength scanning, three key issues must be addressed for accurate FBG interrogation: wavelength overlap between adjacent channels, absolute wavelength calibration, and nonlinear wavelength sweeping within each channel. The 0.3–0.4 nm overlap between adjacent channels ensures gap-free wavelength coverage but results in redundant scanning over the overlapping regions. Moreover, the acquired time-domain signals do not directly provide absolute wavelength information, requiring an accurate and unique time-to-wavelength mapping. Since FBG spectral sampling is performed using equally spaced synchronous trigger signals, a nearly linear wavelength–time relationship is required to ensure uniform wavelength steps between consecutive triggers. However, carrier and thermal effects introduce nonlinear wavelength tuning in DFB lasers, resulting in nonuniform wavelength intervals and consequently errors in FBG spectral reconstruction. Therefore, sweep linearization is required for each DFB channel to establish an accurate and uniform time-to-wavelength mapping.

To address these issues, wavelength calibration is performed using a tunable narrowband filter (TNF) in combination with a 12.5-GHz F-P etalon. The TNF provides an absolute wavelength reference, while the fixed frequency spacing between adjacent F-P transmission peaks provides a dense relative frequency reference for each DFB channel, thereby establishing the complete time-to-wavelength relationship. Previous studies have demonstrated the effectiveness of fixed-FSR F-P transmission peaks for calibrating the time–frequency relationship of high-speed DFB-based swept sources [24].

The sweep nonlinearity is further characterized by extracting the temporal intervals between adjacent F-P transmission peaks. Based on their deviations from the mean interval, the DFB drive current is iteratively adjusted to generate a predistorted current waveform, making the temporal spacing between adjacent F-P peaks nearly uniform and thereby improving the sweep linearity of each channel. Similar iterative current-predistortion approaches have been employed to improve the wavelength-sweep uniformity of DFB lasers. Finally, the wavelength-overlap regions are identified from the calibrated wavelength ranges, and redundant data from one of the adjacent channels are removed. The valid wavelength segments of all channels are then sequentially stitched to obtain a broadband swept output with accurate absolute wavelength calibration, continuous monotonic coverage, and no redundant wavelength mapping, providing the basis for subsequent FBG spectral reconstruction and precise Bragg wavelength interrogation.

## 4. Design and Performance Characterization of the FBG Sensor

**Figure 4:**
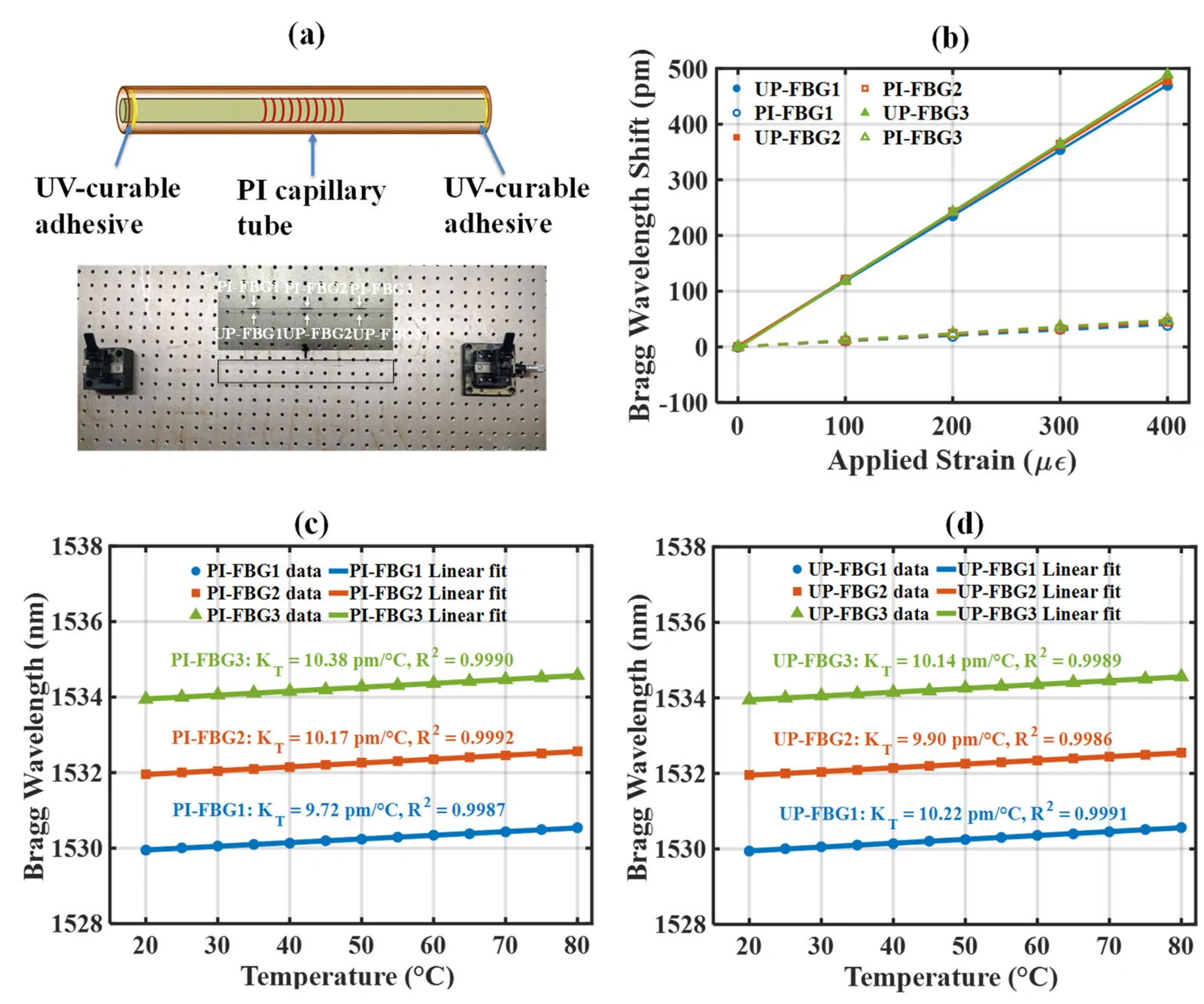
Design and characterization of PI-packaged FBG temperature sensors and temperature calibration of the PI-FBGs and UP-FBGs. (**a**) Schematic of the PI-packaged FBG sensor and experimental setup for comparative strain measurements of PI-packaged and unpackaged FBGs. (**b**) Bragg wavelength shifts of PI-packaged and unpackaged FBGs under different applied strains. (**c**) Temperature calibration results of PI-FBG1–3. (**d**) Temperature calibration results of UP-FBG1–3.

To mitigate the temperature–strain cross-sensitivity caused by surface deformation of lithium-ion batteries during charge–discharge cycling, an FBG temperature sensor with strain-isolation packaging was designed, as illustrated in Figure 4a. The FBGs were fabricated in silica single-mode fibers with a polyimide coating and had a grating length of 10 mm. Their center wavelengths were distributed within 1528–1568 nm, matching the wavelength coverage of the 24-channel REC-DFB laser array. To suppress the influence of battery surface deformation on temperature measurements, the FBG region was enclosed in a PI capillary tube, with both ends of the grating region fixed to the inner wall of the tube using UV-curable adhesive. This configuration maintained the grating region in a mechanically isolated state, allowing external mechanical loads to be primarily borne by the PI capillary and the fixed regions, thereby reducing strain transfer to the grating. The packaged sensor had an outer diameter of less than 1 mm, enabling flexible attachment to specific locations on the battery surface for localized temperature monitoring.

To characterize the temperature response of the packaged sensors, three PI- FBGs located along the same sensing fiber, denoted as PI-FBG1, PI-FBG2, and PI-FBG3, were calibrated. Their initial Bragg wavelengths at 25 °C were approximately 1530, 1532, and 1534 nm, respectively. Prior to calibration, the PI-FBGs were subjected to thermal cycling to release residual stress introduced during packaging. Temperature calibration was subsequently performed over the range of 20–80 °C in increments of 5 °C. At each temperature, the sensors were maintained for 10 min to ensure thermal equilibrium, and three heating–cooling cycles were conducted to evaluate measurement repeatability. At each temperature point, the maximum difference between the largest and smallest Bragg wavelengths measured over the three cycles did not exceed 2.3 pm for any of the three PI-FBGs, confirming good repeatability under the calibration conditions. Figure 4c shows the temperature-dependent Bragg-wavelength variations of PI-FBG1-3. All three PI-FBGs exhibited an approximately linear redshift with increasing temperature. Least-squares linear fitting yielded temperature sensitivities, K_T_ = Δλ_B_ /ΔT, of 9.72, 10.17, and 10.38 pm/°C for FBG1, FBG2, and FBG3, respectively, with corresponding coefficients of determination (R^2^) of 0.9987, 0.9992, and 0.9990. The same temperature-calibration protocol was applied to three unpackaged FBGs, denoted as UP-FBG1, UP-FBG2, and UP-FBG3. Figure 4d shows the temperature-dependent Bragg-wavelength variations of UP-FBG1-3. The UP-FBGs also exhibited an approximately linear redshift with increasing temperature. Least-squares linear fitting yielded temperature sensitivities of 10.14, 9.90, and 10.22 pm/°C for UP-FBG1, UP-FBG2, and UP-FBG3, respectively, with corresponding R2 of 0.9989, 0.9986, and 0.9991. The measured temperatures agreed with the reference temperatures to within ±0.5 °C over the three heating–cooling cycles. The calibration results demonstrate good linearity, stability, and repeatability over the investigated temperature range.

To further quantify the suppression of strain cross-sensitivity provided by the PI packaging, comparative strain experiments were performed using PI-packaged and unpackaged FBGs, as illustrated in Figure 4a. Two optical fibers, each carrying three FBG sensors in series, were fixed axially onto the displacement stages on the left and right sides. The upper row consisted of PI-FBG1, PI-FBG2, and PI-FBG3, while the lower row consisted of UP-FBG1, UP-FBG2, and UP-FBG3. The two rows of gratings were arranged in parallel along the same stretching path to ensure that all gratings experienced identical axial strain during stretching. Both ends of the fibers were clamped by fixtures, and the displacement stage on the right side was equipped with a micrometer head to apply controlled axial displacement to the fibers. The minimum scale of the micrometer head was 10 μm, and the effective fiber length between the two clamping points was 1 m, corresponding to a minimum strain of 10 με. Prior to the experiment, a certain pre-strain was applied to the fibers so that the center wavelengths of all FBGs were set at appropriate operating points. During the experiment, with the ambient temperature kept constant, axial strains of 0, 100, 200, 300, and 400 με were applied sequentially to both fiber groups, and the corresponding Bragg wavelength shifts were recorded. Linear least-squares fitting was performed using *K_ε_* = Δ*λ_B_*/Δ*ε* to determine the strain sensitivity.

The experimental results are shown in Figure 4b. The *K_ε_* of UP-FBG1, UP-FBG2 and UP-FBG3 are approximately 1.18 pm/με, 1.20 pm/με and 1.22 pm/με, with corresponding coefficients of R^2^ of 0.9990, 0.9991, and 0.9989, respectively. For PI-FBG1, PI-FBG2 and PI-FBG3, the strain sensitivities are about 0.10 pm/με, 0.11 pm/με and 0.12 pm/με, respectively, with corresponding R^2^ of 0.9992, 0.9993, and 0.9992, respectively.

To quantitatively evaluate the strain-isolation performance, the strain-suppression ratio *η*_iso_ was defined as:

(6) $$\eta_{\mathrm{iso}}=(1-\frac{K_{\varepsilon ,\mathrm{PI}}}{K_{\varepsilon ,\mathrm{UP}}})\times 100\%,$$

where *K*_ε,PI_ and *K*_ε,UP_ represent the strain sensitivities of the PI-packaged FBG and its corresponding unpackaged FBG, respectively. Based on the fitted results for the PI-FBG1/UP-FBG1, PI-FBG2/UP-FBG2, and PI-FBG3/UP-FBG3 pairs, the strain-suppression ratios of 91.53%, 90.83%, and 90.16%, respectively, were obtained. These results demonstrate that the proposed PI packaging substantially reduces the influence of external mechanical strain on the Bragg wavelength, thereby effectively mitigating temperature–strain cross-sensitivity induced by battery surface deformation and providing a reliable basis for accurate surface-temperature monitoring of lithium-ion batteries.

## 5. Interrogation System and Experimental Results

**Figure 5:**
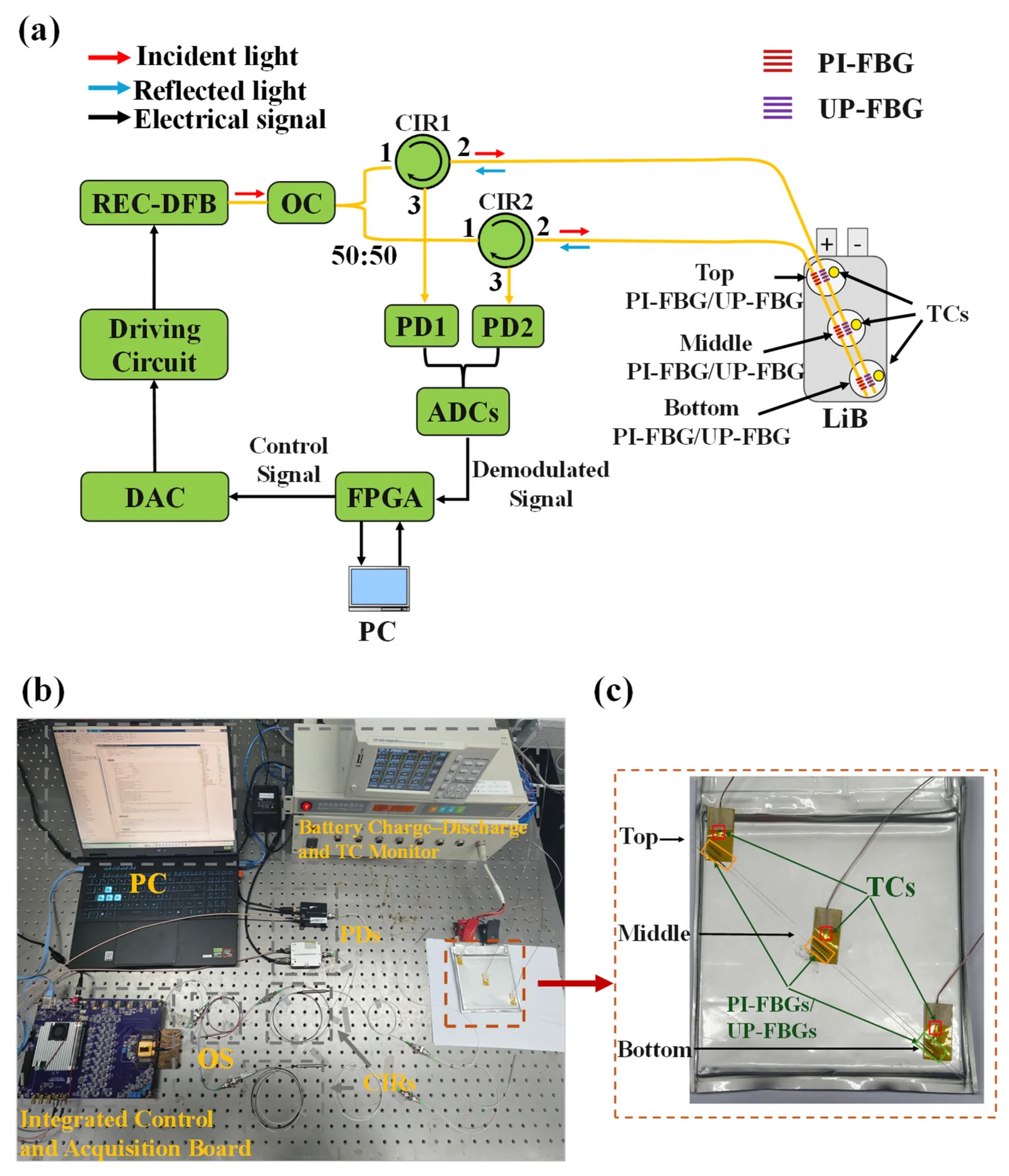
Experimental setup for multi-point temperature monitoring of a lithium-ion battery using the REC-DFB-based high-speed FBG interrogation system. (**a**) Schematic of the experimental system. (**b**) Overview photograph of the experimental setup. (**c**) Close-up photograph of the PI-FBGs, UP-FBGs, and TCs mounted on the battery surface.

To validate the feasibility of the developed REC-DFB high-speed swept source and strain-isolated FBG sensors for real-time temperature monitoring of lithium-ion batteries, a multi-point battery temperature monitoring system was established. As shown in Figure 5, a commercial rechargeable lithium-ion polymer battery with a rated capacity of 1440 mAh, a nominal voltage of 3.7 V, and a maximum charging voltage of 4.3 V was used. The discharge cutoff voltage was 3.0 V. Based on the rated capacity, discharge rates of 0.5 C, 2 C, and 4 C correspond to nominal discharge currents of 0.72, 2.88, and 5.76 A, respectively. To characterize the spatial temperature distribution over the battery surface, FBG sensors were attached to the top, middle, and bottom regions of the battery.

Class 1 T-type thermocouples (TCs) with a temperature measurement junction size of 2 mm × 0.5 mm and a temperature tolerance of ±0.5 °C were placed adjacent to each FBG to provide temperature references. During the experiments, Class 1 K-type sheathed probe thermocouples, with a probe diameter of 1 mm and a temperature tolerance of ±1 °C, were also used for synchronous temperature sampling. Both types of TCs recorded temperature data at a sampling rate of 1 Hz. Zero-phase digital filtering was applied during post-processing to suppress noise while avoiding phase shifts and filtering-induced ringing artifacts in the temperature traces.

The FBGs had a grating length of 10 mm and were separated by 70.0 mm, with an operating temperature range of 0–300 °C without irreversible degradation of their reflection characteristics. The FBGs were attached to the battery surface using UV-curable adhesive. The strain and temperature sensitivity coefficients of both the UP-FBGs and PI-FBGs had been characterized in prior calibration tests.

The REC-DFB interrogation system operates at a repetition rate of 5 kHz for the full stitched 50 nm broadband sweep. The laser wavelength step is 10 pm. Using a Gaussian peak-fitting algorithm, the system achieves a wavelength resolution of 1 pm. Static tests conducted at a constant ambient temperature demonstrate peak-to-peak wavelength fluctuation of 4 pm (0.4 °C in temperature).

Figure 5a illustrates the REC-DFB interrogation system based on a REC-DFB laser array interrogation system for monitoring the temperature of a lithium-ion battery. The driving circuit controls the REC-DFB laser array to perform wavelength scanning. The swept light is split into two paths by a 50:50 fiber coupler (OC) and directed through optical circulators CIR1 and CIR2 to the PI-FBG and UP-FBG sensing branches, respectively. Both types of FBG sensors are positioned at the top, middle, and bottom of the battery (LiB). The reflected light returns along the respective optical paths and is routed by the circulators to photodetectors PD1 and PD2, where it is converted into electrical signals. These signals are acquired by analog-to-digital converters (ADCs) and processed by a field-programmable gate array (FPGA). The demodulated results are transferred to a personal computer (PC), while the FPGA also controls the REC-DFB laser array through a digital-to-analog converter (DAC) and the driving circuit.

Using the calibrated time-to-wavelength mapping, the acquired time-domain signals are reconstructed into FBG reflection spectra, from which the Bragg wavelengths of the individual sensors are extracted. The strain and temperature sensitivities of the UP-FBGs and PI-FBGs were determined in separate calibration experiments. Substituting the wavelength shifts measured by the PI-FBG and UP-FBG measurements into Equation (3) enables the contributions of temperature and strain to be decoupled, thereby yielding the battery-surface temperature change. The PI-FBGs are packaged in PI capillaries to reduce strain transfer caused by deformation of the battery surface, whereas the UP-FBGs provide the response of unpackaged gratings. In the schematic, the red, blue, and black arrows denote the propagation directions of the incident light, reflected light, and electrical signals, respectively.

Figure 5b shows an overall photograph of the experimental setup. Figure 5c provides a close-up view of the FBG sensors and thermocouples mounted on the battery surface, showing the locations of the PI-FBGs, UP-FBGs, and thermocouples.

**Figure 6:**
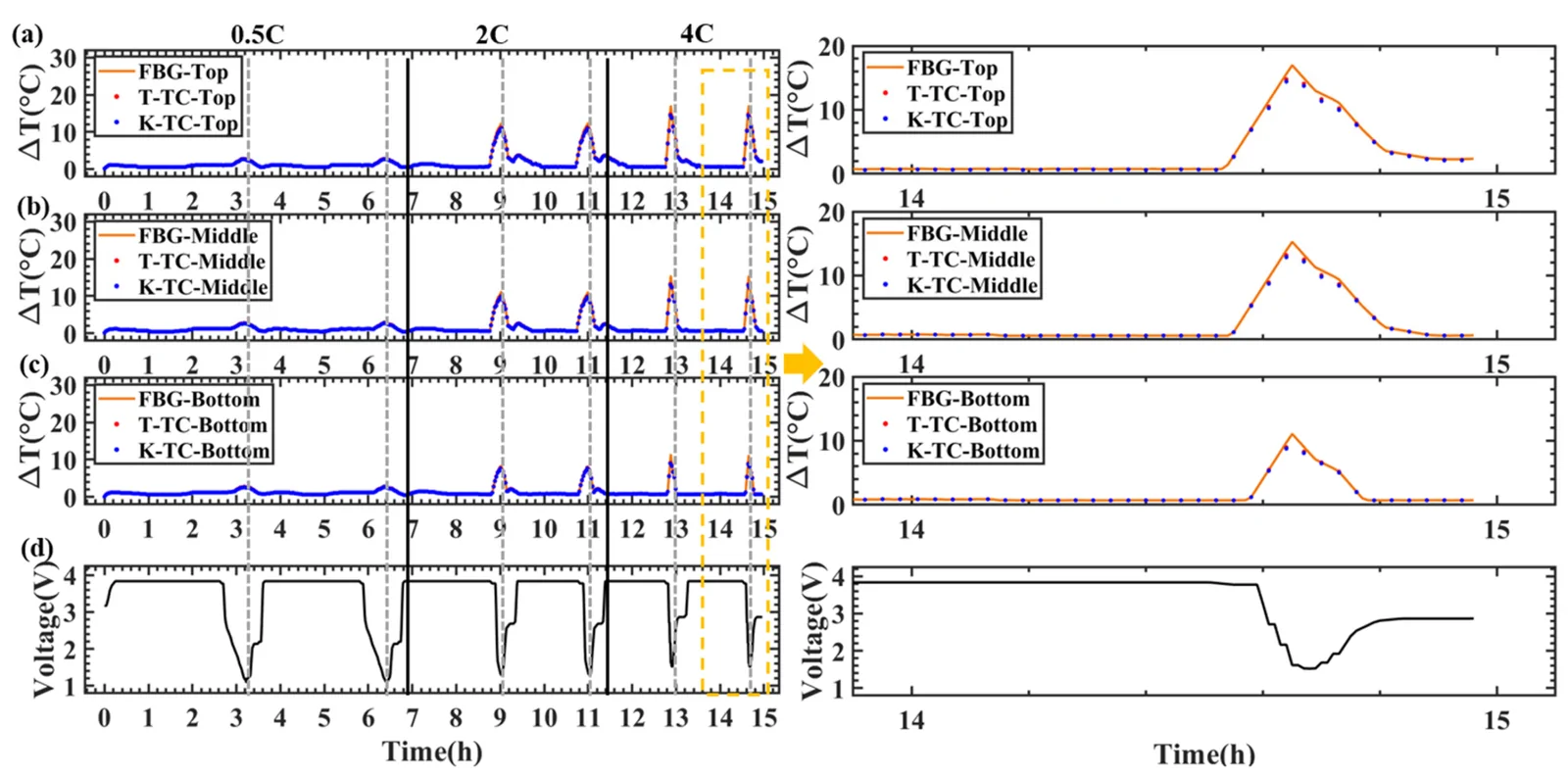
Dynamic temperature monitoring of the lithium-ion battery under different charge–discharge conditions. (**a**–**c**) Temperature responses measured by the FBGs and TCs at the top, middle, and bottom positions, respectively; (**d**) corresponding battery terminal voltage. The enlarged views show the rapid thermal transients during 4 C discharge.

To further evaluate the capability of the proposed system to monitor the dynamic thermal behavior of the battery under different operating conditions, discharge experiments were performed at discharge rates of 0.5 C, 2 C, and 4 C. The battery cycling experiments were conducted at an ambient temperature of approximately 25 °C and were repeated twice. These repetitions were separate from the three heating–cooling cycles used for FBG temperature calibration. The temperature responses measured by the FBGs and T-type thermocouples (TCs) at the top, middle, and bottom positions were recorded simultaneously with the battery terminal voltage. During the experiments, Class 1 K-type sheathed probe thermocouples were additionally employed for synchronous temperature measurements. The recorded temperature and voltage responses are shown in Figure 6.

At low discharge rates of 0.5 C and under relatively slow thermal transients, the maximum temperature rise is approximately 3.1 °C. The peak deviations between FBG-demodulated temperatures and T-type TCs at all three measurement locations are below 0.2 °C, indicating that FBG-derived temperature readings and T-type TCs maintain favorable agreement under relatively slow thermal transients. This result verifies the accuracy of the proposed interrogation system for battery temperature monitoring.

When the discharge rate increases to 2 C, the maximum temperature rises obtained from FBG demodulation at the top, middle, and bottom are approximately 12.41, 11.17 and 8.34 °C, respectively, while the corresponding readings from T-type TCs are about 11.39, 10.27 and 7.85 °C, yielding peak differences of 1.02, 0.90 and 0.49 °C. Further increasing the discharge rate to 4 C, the maximum temperature rises captured by the FBG method reach 17.25, 15.79 and 11.27 °C, whereas those measured by T-type TCs are 14.72, 13.37 and 9.15 °C, with peak differences rising to 2.53, 2.42 and 2.12 °C. These results demonstrate that the peak temperature discrepancy between FBG-demodulated results and TCs gradually grows as the discharge rate and thermal variation rate increase. Nevertheless, FBG-derived temperature readings and TCs still agree well during relatively slow heating and cooling stages; obvious deviations mainly occur during rapid heating and near thermal peaks.

Furthermore, to exclude the influence of intrinsic static measurement errors of the TCs, Class I Type-K sheathed probe TCs were introduced as independent references and used for synchronous measurements with the original Type-T TCs during the experiments.

During fast thermal transients at 4 C, the maximum temperature rises measured by K-type TCs at the top, middle, and bottom are 14.37, 13.07, and 8.95 °C, respectively. The peak differences between the two TC types are only 0.35, 0.30 and 0.20 °C, which are smaller than the 2.1–2.6 °C peak deviations between FBG-demodulated temperatures and TCs. Both types of TCs show similar underestimation of transient temperature, demonstrating that this discrepancy does not mainly originate from static measurement errors or individual sensor variation of a single TC type. These observations suggest that the peak differences observed during fast thermal transients are primarily attributed to the distinct dynamic thermal response characteristics of different measurement approaches. Conventional TCs possess finite junction thermal mass and thermal inertia, which easily induce response lag and temporally smooth transient temperature variations under rapid temperature changes, leading to underestimation of peak temperature. In contrast, the proposed REC-DFB laser array can more fully capture the transient temperature evolution and peaks on the battery surface.

Multi-point FBG measurements also reveal prominent spatial temperature non-uniformity during high-rate discharge. At 4 C, the maximum temperature-rise difference between the top and bottom obtained from FBG demodulation reaches approximately 5.98 °C. Compared with the relatively uniform temperature distribution at low discharge rates, the spatial temperature gradient on the battery surface increases substantially under high-rate discharge. This indicates that single-point temperature measurements may be insufficient to fully characterize the battery thermal state under high thermal loads. The combination of multi-point FBG sensing and high-speed interrogation can simultaneously characterize the spatial temperature distribution and fast thermal transients over the battery surface, providing an effective means to identify local hotspots and thermal anomalies during high-rate operation.

## 6. Discussion

The increasing discrepancy between the FBG and TC measurements at higher discharge rates reflects their different dynamic responses to rapid thermal transients. While good agreement is obtained at 0.5 C, the FBGs record peak temperature rises 2.12–2.53 °C higher than the T-type TCs at 4 C. This difference occurs primarily during rapid heating and near the temperature peaks, suggesting that the thermal inertia of TCs may smooth transient temperature variations. In contrast, the 5 kHz interrogation rate provides sufficiently dense temporal sampling to preserve rapid wavelength variations captured by the FBGs.

The strain-isolated packaging further reduces temperature–strain cross-sensitivity caused by battery deformation. The three tested sensors exhibited similar temperature responses and strain suppression ratios of 90.16–91.53%, providing preliminary evidence of packaging reproducibility. Meanwhile, the 5.98 °C maximum difference in temperature rise between the top and bottom positions at 4 C indicates pronounced spatial thermal nonuniformity, emphasizing the limitation of single-point temperature measurements under high-rate operation. Nevertheless, the present study is limited to three-point surface monitoring of a single cell, and the mechanical strength and long-term reliability of the PI-FBG structure, as well as polarization dependence, were not separately characterized. Polarization-dependent transmission or gain may affect the detected spectral intensity, while FBG birefringence may alter the spectral shape and estimated Bragg wavelength. Further investigations of mechanical reliability, controlled polarization conditions, larger FBG arrays, different battery configurations, and long-term cycling are needed to further evaluate the robustness and scalability of the system.

## 7. Conclusions

A high-speed FBG interrogation system based on a REC-DFB laser array was developed for real-time temperature monitoring of lithium-ion batteries. The multi-wavelength swept source enables broadband and rapid wavelength interrogation, while strain-isolated FBG sensors suppress deformation-induced cross-sensitivity. The system achieves an interrogation rate of 5 kHz and provides temperature measurements consistent with thermocouples under slowly varying conditions. During 4 C discharge, the FBGs capture transient temperature rises 2.12–2.53 °C higher than the thermocouples, demonstrating improved capability for resolving rapid thermal variations. Multi-point measurements further reveal a maximum temperature-rise difference of 5.98 °C across the battery surface. These results demonstrate the potential of the proposed system for high-speed, multi-point thermal monitoring of lithium-ion batteries.

## Figures and Tables

**Table 1:** Wavelength tuning ranges of the 24-channel REC-DFB laser array.

LD	Wavelength Range (nm)	LD	Wavelength Range (nm)
1	1518.023–1520.465	13	1542.693–1545.148
2	1520.109–1522.529	14	1544.803–1547.310
3	1522.176–1524.661	15	1546.888–1549.304
4	1524.287–1526.696	16	1548.917–1551.346
5	1526.266–1528.736	17	1550.937–1553.419
6	1528.376–1530.824	18	1553.042–1555.565
7	1530.471–1532.879	19	1555.135–1557.610
8	1532.505–1534.971	20	1557.190–1559.642
9	1534.590–1537.025	21	1559.256–1561.783
10	1536.638–1539.094	22	1561.365–1563.771
11	1538.653–1541.062	23	1563.400–1565.960
12	1540.670–1543.082	24	1565.556–1567.994

## Data Availability

The data presented in this study are available from the corresponding author upon reasonable request.
